# Intrauterine Twin Discordancy and Partial Postnatal Catch-up Growth in a Girl with a Pathogenic *IGF1R* Mutation

**DOI:** 10.4274/jcrpe.galenos.2019.2018.0236

**Published:** 2019-09-03

**Authors:** Paula Ocaranza, Monique Losekoot, Marie J. E. Walenkamp, Christiaan De Bruin, Jan M. Wit, Veronica Mericq

**Affiliations:** 1 University of Chile Faculty of Medicine, Institute of Maternal and Child Research, Santiago, Chile; 2 Leiden University Medical Center, Department of Clinical Genetics, Leiden, The Netherlands; 3 Emma Children’s Hospital, Amsterdam University Medical Center, Vrije Universiteit Amsterdam, Department of Pediatric Endocrinology, Amsterdam, The Netherlands; 4 Leiden University Medical Center, Department of Pediatrics, Leiden, The Netherlands

**Keywords:** Insulin-like growth factor type-1, insulin-like growth factor type-1 receptor, small for gestational age, postnatal growth, intrauterine discordancy

## Abstract

**Objective:**

Insulin like growth factors-1 (IGF-1) is essential for normal *in utero* and postnatal human growth. It mediates its effects through the IGF-1 receptor (IGF1R), a widely expressed cell surface tyrosine kinase receptor. The aim of the study was to analyze pre- and post-natal growth, clinical features and laboratory findings in a small for gestational age (SGA) girl in whom discordant postnatal growth persisted and her appropriate for gestational age (AGA) brother.

**Methods:**

A girl born with a low weight and length [-2.3 and -2.4 standard deviation (SD) score (SDS), respectively] but borderline low head circumference (-1.6 SD) presented with a height of -1.7 SDS, in contrast to a normal height twin brother (0.0 SDS). IGF-1 resistance was suspected because of elevated serum IGF-1 levels.

**Results:**

Sequencing revealed the presence of a previously described pathogenic heterozygous mutation (p.Glu1050Lys) in the SGA girl which was not present in the parents nor in the AGA twin brother.

**Conclusion:**

The pathogenic *IGF1R* mutation in this girl led to intrauterine growth retardation followed by partial postnatal catch-up growth. Height in mid-childhood was in the lower half of the reference range, but still 1.7 SD shorter than her twin brother.

**What is already known on this topic?**

IGF1R mutations cause prenatal and postnatal decrease in linear growth. This mutation (p.Glu1050Lys) has been tested *in vitro* in fibroblasts which showed a decrease in phosphorylation of STAT5, a protein that, when activated, acts as a transcription factor in the nucleus.

**What this study adds?**

The effect of this mutation on intrauterine growth was tested for the first time in discordant twins. The affected girl’s weight decreased by 36% and her length by 12%. This case highlights that intrauterine twin discordancy can occur in some patients carrying IGF1R mutations.

## Introduction

Insulin like growth factors (IGFs) are essential for intrauterine and postnatal growth and development (1). The mitogenic effects of IGF-1 are mediated through the IGF-1 receptor (IGF1R), a cell surface tyrosine kinase receptor encoded by IGF1R (15q26.3) (2). Synthesized as a single polypeptide precursor, the IGF1R undergoes proteolytic cleavage into α- and β-chains and forms a tetramer (α_2_β_2_), with the extracellular α_2_-subunits involved in ligand binding and the β_2_-subunits carrying intrinsic tyrosine kinase activities (2). Ligand association leads to IGF1R autophosphorylation and activation of multiple downstream signaling pathways (3). This signaling results in fetal somatic growth, whereas postnatal somatic growth is achieved through the synergistic interaction of growth hormone (GH) and IGFs, among other factors (4).

The role of IGFs and their receptors in growth and development was first studied in animal models in which the invalidation of the *Igf1* and *Igf1r* genes in mice causes pre- and post-natal growth retardation (5). Later, genetic studies in short children showed that absent or decreased expression of IGF-1 leads to severe pre- and post-natal growth failure, and microcephaly (6,7,8), while heterozygous (or compound heterozygous hypomorphic) mutations or deletions of *IGF1R* lead to a variable degree of pre- and post-natal growth failure and microcephaly (9,10,11).

Intrauterine growth retardation (IUGR) is not a rare condition and can lead to a small body size for gestational age (SGA) (12). It can be caused by maternal, placental or fetal factors. Approximately 90% of children born SGA show catch-up growth in the first years of life (13,14). In these children no further diagnostic tests are carried out. In children born SGA with persistent short stature multiple genetic causes have been detected (15).

We report a twin girl born SGA with partial catch-up growth, but still 1.7 standard deviation (SD) shorter than her appropriate for gestational age (AGA) born twin brother. Her serum IGF-1 level was unexpectedly elevated, due to a previously described pathogenic mutation in *IGF1R* (c.3148G>A, p.Glu1050Lys).

## Methods

### Subjects

Informed consent was obtained from the family to participate and provide samples (DNA, whole blood), in compliance with the Institutional Ethics Committee at San Borja-Arriarán’s Hospital (Santiago, Chile).

### Sample Procurement

Genomic DNA was isolated from peripheral blood from the patient, her sibling and from both parents. The samples were sent to the Laboratory for Diagnostic Genome Analysis, Department of Clinical Genetics at the Leiden University Medical Center (LUMC) for routine genetic testing of *IGF1R*. Targeted Sanger sequencing of the complete coding region exon 1-21 including intron/exon boundaries (NM_000875.3) was performed as previously reported (10,16). Multiplex ligation-dependent probe amplification (MLPA) assay (MRC Holland kit P217-B2) containing probes for *IGF1R* exon 1-21 was performed for the detection of deletions or duplications (16).

### Statistical Analysis

Comparisons between groups were not performed in this study.

## Results

### Clinical Presentation of the Index Patient

**Figure 1:**
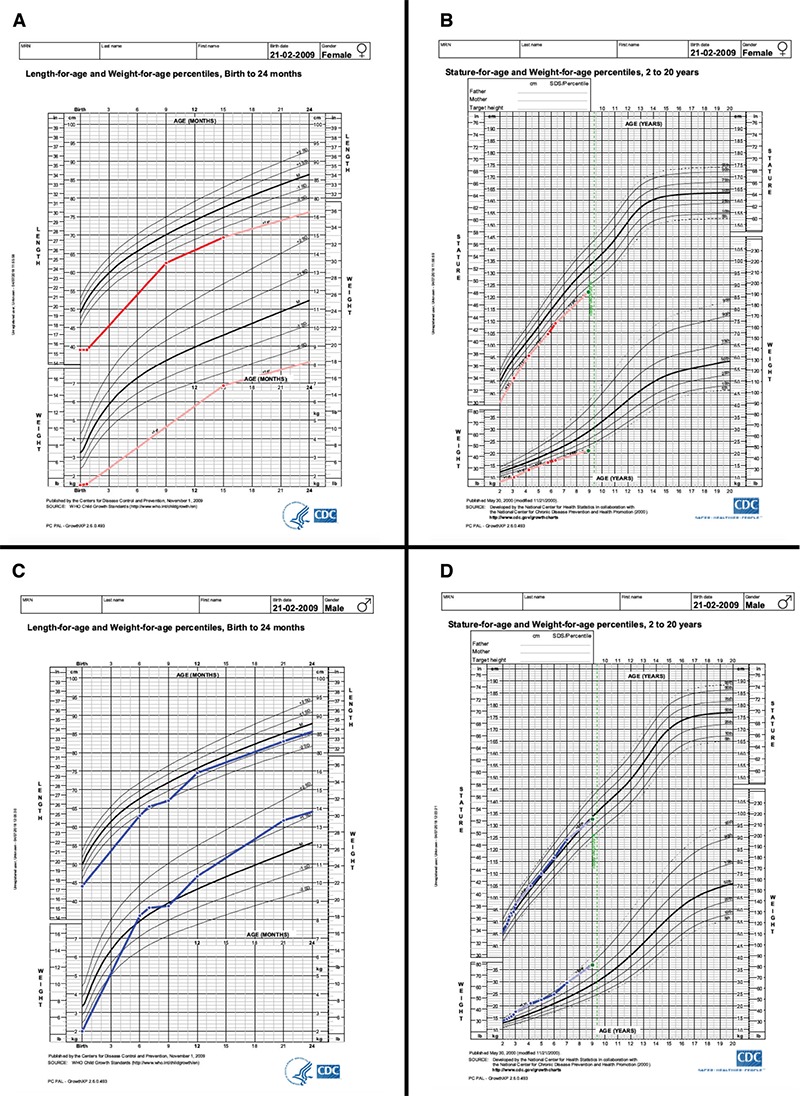
Growth chart of the patient (A) and her twin brother (B). Growth charts of the patient carrying the mutation (C) and (D) growth charts of the normal statured brother

The Chilean female index patient was part of a bichorial biamniotic twin, born after a pregnancy interrupted due to premature membrane rupture and metrorrhagia. The patient showed *in utero* growth discordancy at week 21 and was born SGA at 33 weeks of gestational age, with a birth weight of 1.48 kg [-2.4 SD score (SDS)] (17), a birth length of 39 cm (-2.4 SDS) and a head circumference of 29.5 cm (-1.6 SDS) (Figure 1A). During her first days of life, she was hospitalized for gastric distress. Several episodes of gastro-oesophageal reflux with and without cyanosis were reported after hospitalization.

**Figure 2:**
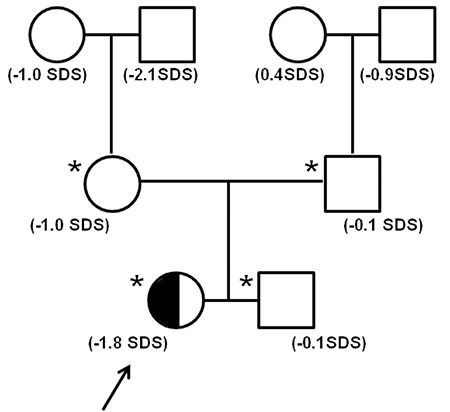
Pedigree of the index patient with the *IGFIR* mutation. Height standard deviation score is indicated in brackets and persons who were checked for the *IGFIR* mutations are indicated (*) SDS: standard deviation score

The parents were not consanguineous. Paternal and maternal heights were 176.9 cm (-0.1 SDS) and 157.9 cm (-1.0 SDS), respectively, with a target height of -0.45 SDS (18). The father reported normally timed puberty and the mother’s pubertal development was slightly delayed (menarche 14 years). Paternal grandfather and -mother had a height of 170 cm (-0.9 SDS) and 165 cm (0.4 SDS), and maternal grandparental heights were 162 cm (-2.1 SDS) and 157 cm (-1.0 SDS), respectively (Figure 2).

The patient was referred to the pediatric endocrine unit for evaluation of short stature at age 1.25 years, because of postnatal growth discordancy with her twin brother (Table 1). Height to arm span ratio was abnormal (≥1.0), weight 6.87 kg (-2.8 SDS for age), weight for height -2.2 SDS (19), and head circumference 44.8 cm (-1.3 SDS). Physical examination revealed normal body proportions and a small midface, mild frontal bossing, a thin upper lip, and mild hypertelorism. Bone age was delayed by three months. A normal female karyotype (46 XX) was found. Serum IGF-1 concentration was high (194 ng/mL; reference range (RR) <131 ng/mL) and IGFBP-3 levels in the upper normal range (3.1 mg/L; RR=1.1-3.6 mg/L). Independent walking was achieved at 1.25 years. Her appetite was poor and selective.

Over the subsequent eight years she visited the clinic several times (Table 1). Psychomotor development was normal. Height remained below -2 SDS up to three years of age and then increased (Figure 1B). Bone age at 3.75 years was delayed but identical to chronological age by 6.33 years. At age 8.92 years she was prepubertal and a small diffuse goiter was noted, confirmed by the finding of a small thyroid cyst at ultrasound. Thyroid function was normal during follow-up. Over the years, her circulating IGF-1 levels and IGFBP-3 concentrations remained high (Table 1).

### The Patient’s Twin Brother

**Figure 3:**
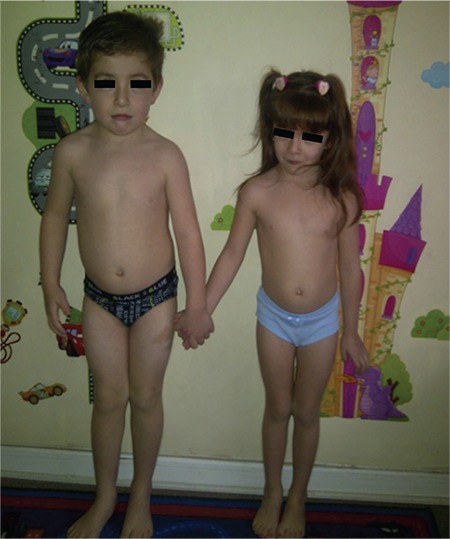
Picture of the twins taken in July 2014

The male twin brother of the index patient was born at 33 weeks of gestational age with a weight of 2.0 kg and length of 44 cm. Growth data are shown in Table 1. At 1.75 years of age, his height was 83 cm and weight was 13.3 kg (Figure 1C). Thereafter his height SDS increased to close to the reference mean (Table 1) and was slightly above conditional target height SDS, and remained stable afterwards (Figure 1D). He has no associated morbidities nor dysmorphic features (Figure 3).

### Genetic Studies

Since the clinical and biochemical characteristics of the index patient were consistent with IGF-1 resistance which could be caused by a deletion or an inactivating mutation in the gene encoding IGF1R, targeted sequencing and MLPA was performed for *IGF1R* on genomic DNA from whole blood from the index patient. Sequence analysis showed a heterozygous nucleotide substitution at position 3148 (c.3148G>A), changing glutamic acid to lysine at position 1050 of the mature IGF1R protein (p.Glu1050Lys). This heterozygous mutation was not encountered in the twin brother nor in either parent. It was confirmed by PP16 analysis that the index patient was the daughter of this couple.

## Discussion

In this study, we report a patient who presented with pre- and post-natal growth retardation resulting from a *de novo* heterozygous *IGF1R* mutation in exon 16 (c.3148G>A, p.Glu1050Lys). Substitution of this highly conserved amino acid residue, located in the intracellular tyrosine kinase domain, is associated with a change in charge of the amino acid and *in silico* analysis predicts inactivation of the IGF1R leading to a partial resistance to IGF-1. This mutation was not identified in the patient´s twin AGA born normal-statured brother nor in other family members.

Fetal growth and development are influenced by maternal, placental and fetal factors (1). A variety of maternal and utero-placental factors may constrain the growth of the fetus. In this interesting experiment of nature the role of maternal and placental factors are well controlled and separated from the role of fetal factors. A series of elegant investigations in mice, complemented by case studies in humans, have convincingly demonstrated the critical role of the IGF system in pre- and post-natal growth (5). Targeted disruption of the gene encoding Igf-2 in mice resulted in a 40 percent reduction in fetal growth with normal postnatal growth, demonstrating the important role of IGF-2 in intrauterine growth. Disruption of the gene for Igf-1 led to a similar decrease in birth weight but also led to persistent postnatal growth failure. Furthermore, deletion of the gene encoding Igf1r, which mediates the growth-promoting actions of both Igfs, resulted in birth weights that were only 45 percent of normal and these mice generally died within hours after birth from respiratory insufficiency due to muscular hypoplasia (5). The relevance of these findings for human growth was supported by reports on humans. Homozygous mutations of *IGF-1* were found in a few patients presenting with severe pre- and post-natal growth failure, microcephaly and deafness (6,7). Several reports have been published of patients with IGF-1 resistance due to molecular defects in the *IGF1R* who present with a variable degree of pre-and post-natal growth retardation (9).

Short stature is a common problem confronting pediatric endocrinologists. After exclusion of systemic or skeletal diseases or overt hormonal deficiencies, clinicians are often unable to provide a definitive diagnosis for the etiology of an individual patient’s short stature. An important clue for the cause of short stature is to register whether prenatal growth was normal or reduced. We suspected a mutation within the IGF-1 signaling cascade because of the persistent short stature in our patient and the high IGF-1 levels. Our hypothesis led us to the detection of a *de novo* heterozygous mutation of *IGF1R* in exon 16, resulting in the replacement of a Glu residue at position 1050 by a Lys residue. So far, mutations in *IGF1R* were almost always reported to result in IUGR, and postnatal catch-up growth had not been documented. Aberrant *IGF1R* expression is described to lead to *IGF1R* haploinsufficiency (20,21), disturbed processing of the proreceptor (22,23), decreased ligand binding (24), abrogated IGF1R tyrosine kinase activity and reduced receptor autophosphorylation (10,25,26).

In line with the previously reported adult patient (with a birth weight and length of -2.1 and -0.3 SDS, respectively, and a height SDS of -3.3 at presentation, and an adult head circumference SDS of -3.0), the mutation led to a clinically significant prenatal and postnatal growth failure, though postnatal growth of our patient is less affected compared to almost all cases with *IGF1R* haploinsufficiency described to date. This mutation was also associated with microcephaly, but it did not affect intellectual development. Our patient was reported to have feeding problems during the first year of life and poor appetite, which previously has been associated with the same and other *IGF1R* mutations (10). This mutation was not present in her twin brother and parents, who all have normal stature. Our results provide strong evidence that this variant is likely to be the underlying cause of the IUGR and mild postnatal short stature observed in this patient.

Most of the *IGF1R* mutations have been described in children born SGA. The first human IGF1R defects were described by Abuzzahab et al (9) in 2003 and only a few compound heterozygous cases have been described thereafter (9,27). Most of the described cases are heterozygous carriers of *IGF1R* mutations (10,20,21,22,23,25,26,28,29,30,31,32,33,34). To date only two single patients carrying a homozygous mutation have been described (35,36). The phenotype is variable, presumably depending on the impact of the mutation on the function of the IGF1R. The most common feature described in the reported patients included IUGR (11,37), postnatal growth failure and microcephaly (11,37,38).

### Study Limitations

The affected Glu residue at position 1050, is located in the strongly conserved serine-threonine/tyrosine-protein kinase catalytic domain. A study limitation was the absence of functional studies, as fibroblasts from skin biopsies were not available. However, functional analysis of fibroblasts from a previously described patient with the same mutation showed a marked reduction of autophosphorylation of the IGF1R and of activation of PKB/Akt upon a challenge with IGF-1. Furthermore, [^3^H]thymidine incorporation in that patient’s cells after a challenge with a dose range of IGF-1 in comparison with a panel of fibroblast cultures of eight non-growth-retarded individuals (controls) showed a 50% reduction (10).

## Conclusion

In conclusion, we describe a discordant pair of twins in whom the effect of this *IGF1R* mutation in the context of a similar intrauterine environment is unmasked. This clinical observation shows that while it is assumed that most patients carrying IGF1R mutations remain short postnatally, partial catch-up growth can occur, possibly related to increased GH and IGF-1 secretion.

## Figures and Tables

**Table 1:**
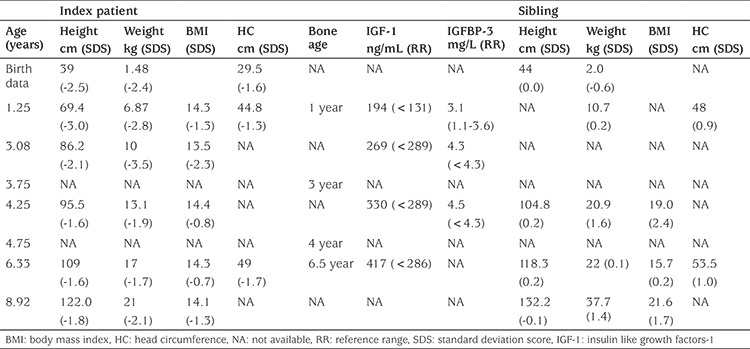
Clinical and biochemical characteristics of the index patient and her twin brother
